# Novel non-synonymous and synonymous gene variants of *SRD5A2* in patients with 46,XY-DSD and DSD-free subjects

**DOI:** 10.1371/journal.pone.0316497

**Published:** 2025-03-05

**Authors:** Luis Ramos

**Affiliations:** Department of Reproductive Biology, Instituto Nacional de Ciencias Médicas y Nutrición Salvador Zubirán, México City, México; Sichuan University, CHINA

## Abstract

*SRD5A2* gene variants are associated with deficiency of steroid 5α-reductase type 2, which is an autosomal recessive disorder of sex development (DSD) present in 46,XY males with ambiguous genitalia. To determine the causality of the disorder, this study involved genetic screening of *SRD5A2* in six unrelated patients with this condition. Polymerase chain reaction (PCR) assays excluded large duplications, insertions, or deletions, while bidirectional Sanger sequencing identified 15 single-nucleotide variants (SNVs), six patients with 46,XY-DSD carrying pathogenic non-synonymous SNVs (nsSNVs), and three subjects who were DSD-free with novel synonymous SNVs (sSNVs). Genomic outcomes showed that 9 non-synonymous coding SNVs are linked to patients with *SRD5A2*-associated steroid 5α-reductase type 2 deficiency (c.169G > C: p.E57Q; c.145G > A: p.A49T/c.686T > C: p.F229S; c.100G > A: p.G34R/c.344G > A: p.G115D; c.591G > T: p.E197D; c.92C > T: p.S31F/c.481A > C: p.I161L (a novel missense variant; *K*_*m*_,_*app*_ =  1.19 ±  0.1 μM, *V*_*max*_,_*app*_ =  688 ±  145.8 pmol/mg P/min); c.686T > C: p.F229S). This analysis also highlighted 2 non-disease-causing sSNVs in three DSD-free subjects (c.243G > T: p.T81 = ; c.594C > T: p.I198=). These silent mutations or sSNVs in the *SRD5A2* gene have no functional consequences and might not be involved in steroid 5α-reductase 2 deﬁciency. The identification of these sSNVs in both healthy controls and patients might suggest natural genetic variability with a very low allele frequency in the Mexican population. Furthermore, these findings indicated that nsSNVs in the *SRD5A2* gene altered normal development of external male genitalia, supporting their pathogenicity.

## Introduction

Human steroid 5α-reductase type 2 (EC 1.3.99.5; SRD5A2) is a nicotinamide adenine dinucleotide phosphate (NADPH)-dependent enzyme that reduces the double bond at the four to five position in C_19_ (testosterone and androstenedione) and C_21_ (progesterone and corticosterone) 3-keto steroids [1–5]. During embryogenesis, SRD5A2 induces midline fusion and is responsible for elongation and enlargement of the urogenital tubercle and the urogenital folds. It is also responsible for differentiation of the external genitalia, male urethra, penis, scrotum, and prostate gland and induces testicular descent. At puberty, SRD5A2 induces deepening of the voice, development of muscle mass, and virilization of external genitalia. Clinical studies also suggest that SRD5A2 plays a role in the development of facial and body hair [5,6].

Pathogenic variants in the human *SRD5A2* gene (chromosome: 2p23.1; transcript: NM_000348.4; region 2: 31747550–31806136; 5 exons and 4 introns, which comprises and open reading frame of 762 base pairs; accessed on March 2, 2023) are associated with deficiency of steroid 5α-reductase type 2 (OMIM: #607306 and OMIM: #264600). This deficiency is an autosomal recessive hereditary disease and genetic steroid disorder caused by homozygous or compound heterozygous loss-of-function mutations, which result in impaired conversion of testosterone (T; 17β-hydroxy-4-androsten-3-one) to 5α-dihydrotestosterone (DHT; 17β-hydroxy-5α-androstan-3-one). Inactivating gene variants of *SRD5A2* result in a broad clinical spectrum of masculinization defects [5,7–11].

In individuals with the 46,XY karyotype, this disorder of sex development (46,XY-DSD) is phenotypically characterized by complete female genitalia with normal Wolffian structures or undervirilization characteristics, such as an isolated micropenis, bifid scrotum, pseudovaginal perineoscrotal hypospadias, cryptorchid testes (gonads may be located abdominally in the inguinal canal or scrotum), prostate hypoplasia, impaired spermatogenesis, absence of facial and body hair, acne, and in exceptional cases, gynecomastia [11–14]. Human congenital and hereditary disorders associated with the SRD5A2 gene provide genetic evidence of a role of missense mutations or non-synonymous single-nucleotide variants (nsSNV, the majority of which are common genetic variants); these disorders also highlight this gene’s biological role in sex determination and differentiation during embryonic development. However, copy number variations (CNVs) are considered to be rare genetic variants. To date, more than 100 mutations in *SRD5A2* that cause steroid 5α-reductase type 2 deficiency have been reported, and the enzymatic kinetics of this genetic disorder are known in very few clinical cases [5].

In the coding region, different genetic and molecular reports have shown that 2 well-known *SRD5A2* gene polymorphisms or nsSNVs (rs9282858: c.145G>A, p.A49T; rs523349: c.265C>G, p.L89V; accessed on March 2, 2023) might be related to benign prostatic hyperplasia and possibly prostate cancer [15–18]. In Korean men with androgenetic alopecia, these polymorphisms were not associated with clinical types of baldness [19]. The aim of this study was to identify mutations or gene variants that are responsible for steroid 5α-reductase type 2 deficiency in patients with 46,XY-DSD and ambiguous genitalia in a Mexican population. Healthy, unrelated controls were selected to validate pathogenic and non-pathogenic SNVs. This study reports 15 gene variants in *SRD5A2* from patients with 46,XY-DSD and steroid 5α-reductase type 2 deficiency and in DSD-free subjects. Based on the results, 9 variants were classified as nsSNV in patients with *SRD5A2*-associated 46,XY-DSD; 3 were nsSNV and 3 were synonymous SNVs (sSNVs) in DSD-free subjects.

## Materials and methods

### Patients and participants

The patients investigated included six unrelated males with 46,XY-DSD and steroid 5α-reductase type 2 deficiency. The clinical diagnosis of the disorder was based on physical examination. At admission, phenotypic examination revealed microphallus, hypospadias, cryptorchidism, pseudovagina, and a lack of mammary glands or axillary/pubic hair. The patients (P1–P6) did not present other diseases or endocrine disorders. All informed consent authorized the study of genomic DNA (gDNA) only, so any hormonal studies were excluded.

The study also included three hundred healthy unrelated controls (N =  300; six unrelated healthy subjects as controls showed a SNVs, C1–C6). The study of *SRD5A2* genetic variants located in controls was carried out in 150 men and 150 women. Subjects were aged 18–42 years. The control subjects and positive fertility and normal sexual development and (S1 Table) were recruited from the Hormonal Biochemistry Laboratory, Department of Reproductive Biology, Instituto Nacional de Ciencias Médicas y Nutrición Salvador Zubirán (INCMNSZ). All participants had Mexican ancestry. The sample size (N =  300) was calculated using G * power software (version 3.1.9.7 Heinrich-Heine-Universitat Dusseldorf, Dusseldorf, Germany), targeting a 90% power with a 5.0% significance level (https://stats.oarc.ucla.edu/other/gpower/). Their ancestry was estimated using local ancestry-history records. Written informed consent was obtained from all participants before sample collection, between September 2021 and September 2022. The study was conducted in accordance with the Declaration of Helsinki and was approved by the ethics committee of INCMNSZ (BRE-2613-18-20-1; September 2021–2022).

### gDNA isolation

The participants provided 10 ml of peripheral blood. The method of gDNA isolation from leukocytes has been described previously in detail [20]. gDNA was quantified using a Beckman spectrophotometer (DU 650, Fullerton, CA, USA), and A260/280 values between 1.8 and 2.0 were was ensured. The final concentration of each gDNA sample was calculated as 300 ng/ µ L. For long-term storage, all gDNA was kept at − 20 °C until analysis.

### Gene amplification of human *SRD5A2*

Exons 1–5 and their adjacent introns of the *SRD5A2* gene (gene ID: 6716; transcript: NM_000348.4) were individually amplified by polymerase chain reaction (PCR) from 300 ng/ µ L of gDNA. The primers and its conditions were stablished according to a predefined protocol [5]. The PCR reactions included 4 µ L of 5X GoTaq flexi buffer (Promega, Madison, WI, USA), 0.5 µ L of each forward and reverse oligonucleotide (20 µ M for each), 0.5 µ L of dNTP (10 mM; Promega, Madison, WI, USA), 0.1 µ L of GoTaq DNA polymerase (5 u/ µ L; Promega, Madison, WI, USA), 1.5–2.5 mM of MgCl_2_ (MgCl_2_ solution 25 mM, Promega, Madison, WI, USA), 1.0 µ L of dimethyl sulfoxide (DMSO), 300 ng of gDNA, and nuclease-free water up to a volume of 20 µ L. Oligonucleotides and thermal cycling conditions were the same as described previously [5].

To verify the amplifications, 1% TBE-agarose gels containing 0.5 µ L/100 mL of ethidium bromide were used. Exon size was determined by comparison to a 100-base-pair (bp) molecular weight marker (MWM, GeneRuler 100 bp DNA Ladder, Thermo Scientific, Vilnius, Lithuania). Electrophoresis was carried out at 100 V for 1 h. Exonic ampliﬁcations were observed using a UV transilluminator (Molecular Imager Gel Doc XR System, BioRad Laboratories, Hercules, CA, USA). Exon isolation from agarose gels was carried out via the centrifugation protocol of Omega Bio-tek’s E.Z.N.A.® Gel Extraction kit according to the manufacturer’s instructions (Omega Bio-Tek, Inc., Norcross,GA, USA).

### Genetic analysis of human *SRD5A2*

Bidirectional Sanger-sequencing was performed for the screening of *SRD5A2* gene variants using a BigDye Terminator v3.1 Cycle Sequencing kit (Thermo Fisher Scientific, Applied Biosystems, Austin, TX, USA) and the corresponding primer set, as reported previously [5,20]. First, 10 ng/ µ L of purified PCR products were mixed with 2 µ L of BigDye Terminator Sequencing RR-100, 1 µ L of BigDye Terminator Sequencing 5X Sequencing Buffer, 1 µ L of 20 µ M oligonucleotide, and 5 µ L of RNase-free water. Incubation was performed at 96 °C for 1 min (Veriti 96-well Thermal Cycler), followed by 35 cycles of denaturing, annealing, and elongation at 96 °C for 10 s, 50 °C for 5 s, and 60 °C for 4 min. Then, the PCR-products were purified by adding a mixture of 45 µ L of SAM buffer and 10 µ L of BigDye Xterminator bead solution.

The sequencing reaction mixtures were thoroughly shaken for 30 min (2000 rpm; BV1000 Vortex Mixer, Edison, NJ, USA) and centrifuged at 1000 ×  g for 2 min at room temperature. The samples were then analyzed by following the manufacturer’s instructions (Applied Biosystems, Foster City, CA, USA) and subjected to capillary electrophoresis using the run module KB_310POP6_BDTv3_36Rapid (temperature: 50 °C; injection voltage: 15 kV; injection time: 15 s; 5 to 8 µ A) on an ABI PRISM 310 Automated sequencer (Applied Biosystems, Foster City, CA, USA). Sequencing reactions for each sample were performed two times independently. Data analysis and interpretation of dideoxy sequencing reactions were performed using Chromas software.

### Mutagenesis and enzyme activity of wild-type and mutant p.I161L-SRD5A2

Human embryonic kidney 293 (HEK293) cells were transfected with the pCMV6-XL4 plasmid (OriGene Technologies Inc., Rockville, MD, USA), which contained the full-length human *SRD5A2* cDNA (clone #SC119922, Rockville, MD, USA). The plasmid was mutated using two mutagenic primers (5´-gcgcaatatatagtcactatgaa**g**gtttattcccattcccaaaataa-3´ and 5´- ttattttgggaatgggaataaac**c**ttcatagtgactatatattgcgc-3´) according to QuikChange Primer Program (https://www.agilent.com/store/primerDesignProgram.jsp) and synthesized by Integrated DNA Technologies (IDT, Coralville, CA, USA). A nucleotide-substituted mutant was produced using the QuikChange Lightning Site-Directed Mutagenesis kit (Agilent Technologies, Santa Clara, CA, USA) according to previously established conditions [5]. HEK293 cells were cultured, transfected, and incubated at 37 °C for 24 h before the assays in three independent biological experiments. 5α-reductase activity was calculated by measuring the rate of formation of [^3^H]-DHT from [^3^H]-T. The protein (P) concentration was quantiﬁed using protein assays with Coomassie Blue (Bradford) as described previously [5]. The apparent kinetic constants (*K*_*mapp*_ and *V*_*maxapp*_) for each variant were presented as means and standard deviations from three parallel experiments. *V*_*max*_ (maximum initial velocity) was the maximum rate of product formation by an enzyme. *K*_*m*_ (the Michaelis-Menten constant) is the substrate concentration at which half-maximal product formation is achieved by an enzyme. *V*_*max*_/*K*_*m*_ is the catalytic eﬃciency for human steroid 5α-reductase type 2.

### Pathogenic mutation prediction and three-dimensional (3D) structure

The pathogenic impact was predicted in human mutated SRD5A2 protein using the PROVEAN program (neutral eﬀect with a score above the threshold or deleterious eﬀect with a score equal to or below a predeﬁned threshold, Protein Variation Eﬀect Analyzer; http://provean.jcvi.org/seq_submit.php) and the PolyPhen-2 program (benign with a score of 0.0, possibly damaging with a score of 0.5, or probably damaging with a score of 1.0, Polymorphism Phenotyping; http://genetics.bwh.harvard.edu/pph2/). The Robetta protein prediction server (https://robetta.bakerlab.org/) was used to generate an enzyme structure, and the wild type (WT) and mutated protein structures were visualized with PyMOL software (http://www.pymol.org/).

## Results

### Exonic analysis

**Fig 1:**
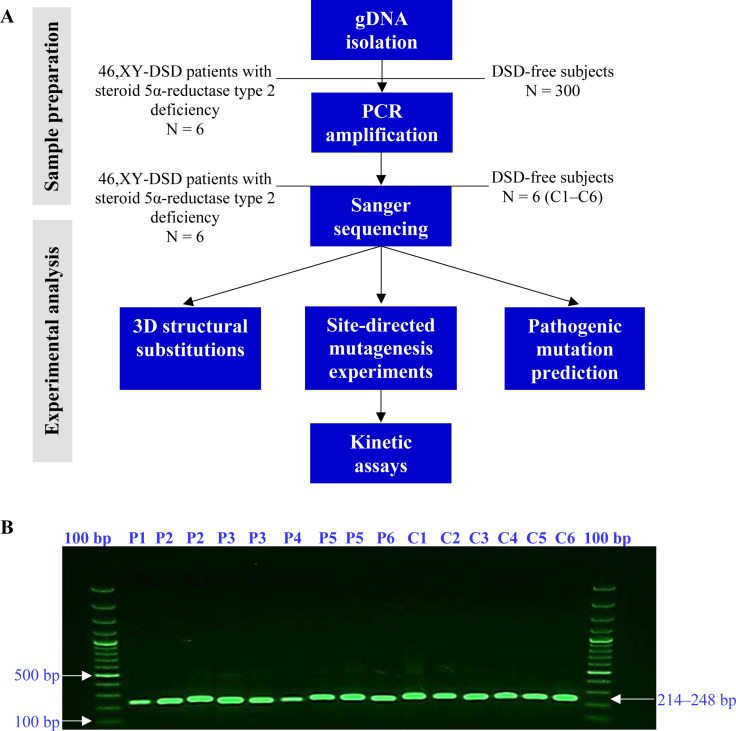
Electrophoretic analysis of exons 1–4 of the human *SRD5A2* gene from TBE-agarose gel. (A) Gene screening and experimental flowchart. This flowchart shows the steps used to process gDNA and screen for relevant exons in the *SRD5A2* gene. (B) The amplification shows the exons of patients (P1–P6) with steroid 5α-reductase type 2 deficiency compared to healthy controls (C1–C6, only exon 1). A specific band of approximately 200–250 bp (blue arrow) was detected in the samples. The white arrows indicate 500 bp, and 100 bp indicates the molecular weight marker (100–1500 bp).

A flowchart of the study design is displayed in Fig 1A. The human *SRD5A2* gene was analyzed in six patients (P1–P6) with a clinical diagnosis of 46,XY-DSD steroid 5α-reductase type 2 deficiency and in six unrelated healthy subjects as controls (C1–C6) who were DSD-free. PCR assays of coding exons and its exon–intron boundaries of the *SRD5A2* gene excluded large deletions, large duplications, large insertions, or large insertions/deletions. Therefore, all exons had an expected/specific molecular size of 200–250 bp (Fig 1B).

### Identification of *SRD5A2* variants

**Fig 2:**
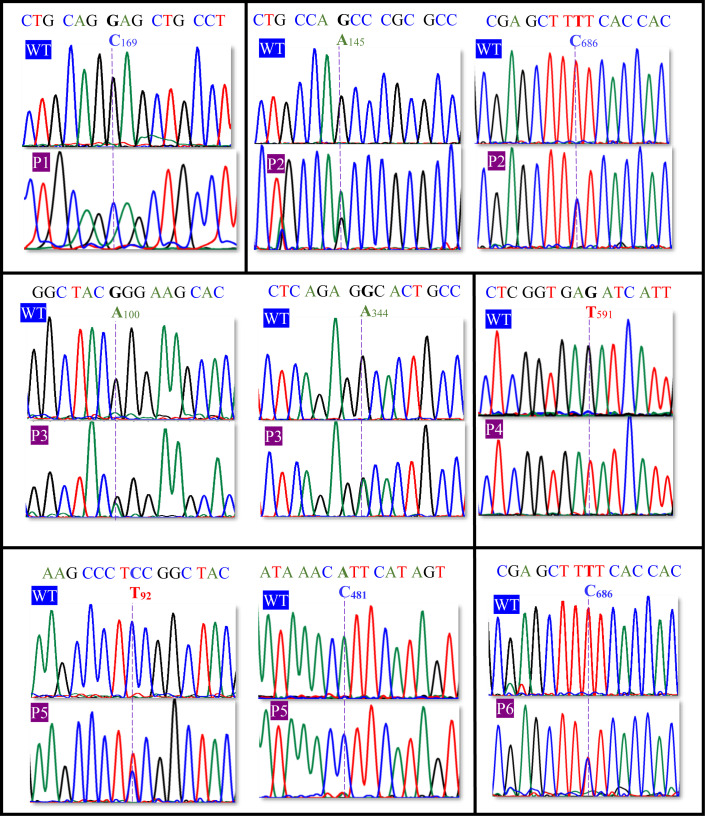
Wild-type (WT) sequences and SNV of human *SRD5A2* gene from patients (P1–P6) with 46,XY-DSD associated with steroid 5 α-reductase type 2 deficiency. DNA sequencing identified 9 nsSNVs (blue letters) at specific nucleotide positions (blue numbers) of cDNA with accession number NM_000348.4 (c.169G > C: p.E57Q; c.145G > A: p.A49T; c.686T > C: p.F229S; c.100G > A: p.G34R; c.344G > A: p.G115D; c.591G > T: p.E197D; c.92C > T: p.S31F; c.481A > C: p.I161L (a novel missense variant); c.686T > C: p.F229S). The dotted lines indicate the sites of nsSNVs.

**Fig 3:**
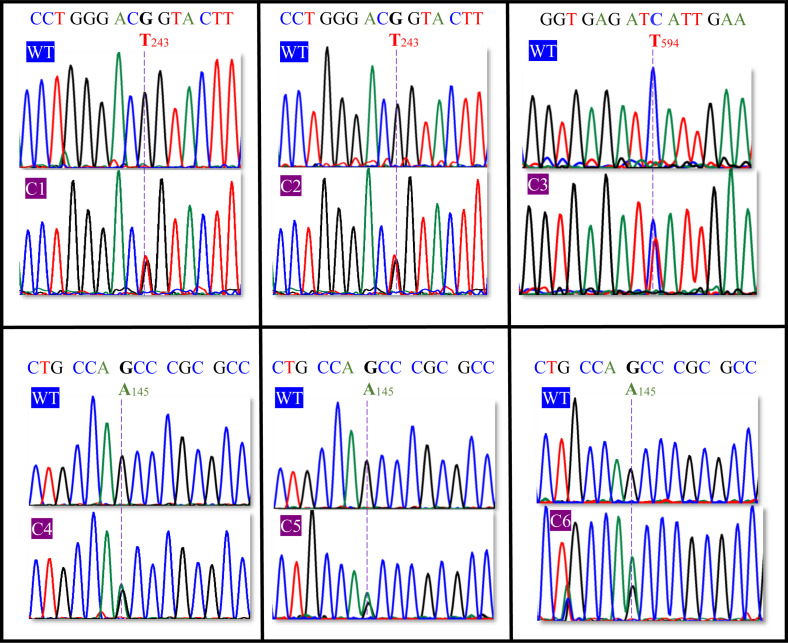
Sanger sequencing identified 6 SNVs (blue letters) at specific nucleotide positions (blue numbers) of cDNA-SRD5A2 with accession number NM_000348.4. Gene variants (C1–C6) obtained from the controls were compared using WT sequences. The dotted lines indicate the sites of sSNVs (C1–C3 with c.243G > T: p.T81 = ; c.594C > T: p.I198=) and nsSNV (C4–C6 with c.145G > A: p.A49T) from DSD-free subjects or unrelated healthy subjects.

Sanger sequencing analysis of the PCR amplicons revealed an association between the 6 patients with steroid 5α-reductase type 2 deficiency and 9 heterozygous non-synonymous gene variants of *SRD5A2* (Fig 2, Table 1). Genomic outcomes showed three patients with compound heterozygous gene variants (P2 =  c.145G > A (p.A49T), c.686T > C (p.F229S); P3 =  c.100G > A (p.G34R), c.344G > A (p.G115D); P5 =  c.92C > T (p.S31P), c.481A > C (p.I161L), one patient with a heterozygous gene variant (P6 =  c.686T > C (p.F229S), and two patients with homozygous conditions (P1 =  c.169G > C: p.E57Q; P4 =  c.591G > T (p.E197D). When single-nucleotide gene variants of P1–P6 were compared with those of unrelated healthy subjects (C1–C6), gDNA analysis detected different single-nucleotide substitutions (Fig 3, Table 1). In 3 controls, 2 different heterozygous variants encoding for synonymous single-nucleotide gene variants were identified (C1 and C2 =  c.243G > T (p.T81=); C3 =  c.594C > T (p.I198=). Direct sequencing of the *SRD5A2* gene revealed 1 heterozygous non-synonymous substitution in 3 unrelated healthy subjects (C4, C5, and C6 =  c.145G > A (p.A49T). These gene variants were analyzed in a group of 300 unrelated healthy subjects who served as controls.

**Table 1:** Genetic analysis of the *SRD5A2* gene from 46,XY-DSD patients with steroid 5α-reductase type 2 deficiency and DSD-free subjects.

	Age years	Exon	Codon	SNV	Variant	PolyPhen-2 Score	PROVEAN Score	Condition	Comments
P1	18	1	**G**AG-**C**AG	c.169G > C	p.E57Q	Pb Dam_0.983	Deleterious − 2.856	Homozygous nsSNV	Penoscrotal hypospadias, no breast development or Müllerian ducts
P2	32	1 4	**G**CC-**A**CC T**T**T-T**C**T	c.145G > A c.686T > C	p.A49T p.F229S	Pb Dam_0.983 Pb Dam_0.996	Deleterious − 2.856 Neutral − 1.888	Compound heterozygous nsSNV	Perineal hypospadias, unilateral cryptorchidism
P3	8	1 2	**G**GG-**A**GG G**G**C-G**A**C	c.100G > A c.344G > A	p.G34R p.G115D	Pb Dam_1.000 Pb Dam_0.999	Deleterious − 5.337 Deleterious − 2.929	Compound heterozygous nsSNV	Pseudovagina, microphallus, inguinal testis. no labia majora
P4	18	4	GA**G**-GA**T**	c.591G > T	p.E197D	Pb Dam_1.000	Deleterious − 2.906	Homozygous nsSNV	Microphallus and inguinal cryptorchidism
P5	17	1 3	T**C**C-T**T**C **A**TT-**C**TT	c.92C > T c.481A > C	p.S31F p.I161L	Pos Dam_0.839 Pos Dam_0.748	Neutral − 1.257 Neutral − 1.562	Compound heterozygous Novel nsSNV	Perineal hypospadias, bilateral testes, microphallus, no breast development *K*_*m,app*_ = 1.19 ± 0.1 µ M; *V*_*max,app*_ = 688 ± 145.8 pmol/mg P/min
P6	24	4	T**T**T-T**C**T	c.686T > C	p.F229S	Pb Dam_0.996	Neutral − 1.888	Heterozygous nsSNV	CAIS patient with incomplete regression of Müllerian ducts and a p.G743E mutation in *NR3C4* gene (Chavez et al., 2001)
									
C1	24	1	AC**G**-AC**T**	c.243G > T	p.T81 =	Neutral 0.000	Neutral 0.000	Novel heterozygous sSNV	^*^Normal XX female
C2	18	1	AC**G**-AC**T**	c.243G > T	p.T81 =	Neutral 0.000	Neutral 0.000	Novel heterozygous sSNV	^*^Normal XY male
C3	32	4	AT**C**-AT**T**	c.594C > T	p.I198 =	Neutral 0.000	Neutral 0.000	Novel heterozygous sSNV	^*^Normal XY male
									
C4	40	1	**G**CC-**A**CC	c.145G > A	p.A49T	Pb Dam_0.983	Deleterious − 2.856	Heterozygous nsSNV	^*^Normal XY male
C5	42	1	**G**CC-**A**CC	c.145G > A	p.A49T	Pb Dam_0.983	Deleterious − 2.856	Heterozygous nsSNV	^*^Normal XY male
C6	28	1	**G**CC-**A**CC	c.145G > A	p.A49T	Pb Dam_0.983	Deleterious − 2.856	Heterozygous nsSNV	^*^Normal XX female

* Healthy males and females served as controls (C1–C6).

### Functional assays of enzymatic kinetics of WT and mutated p.I161L variant

**Fig 4:**
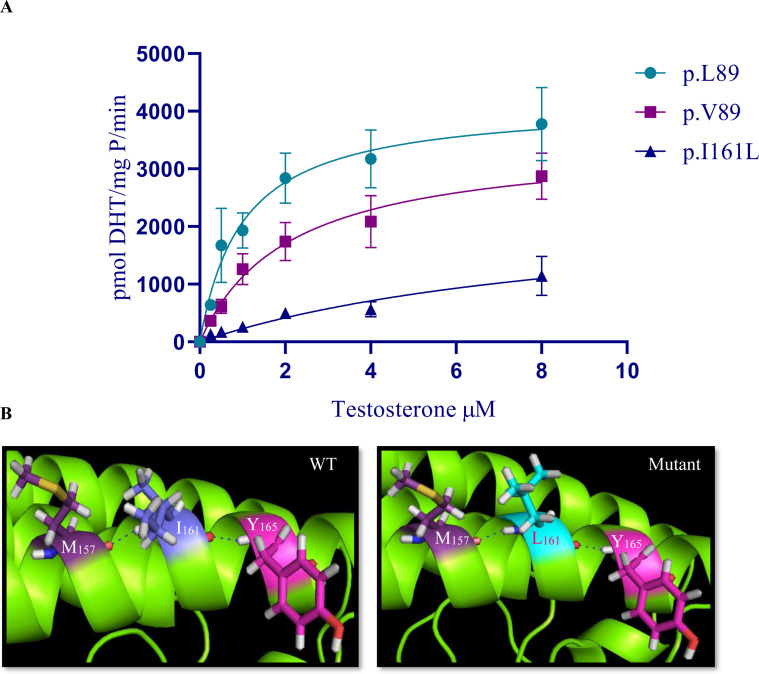
*In vitro* and *in silico* functional/structural analysis studies of SRD5A2 protein from patients with 46,XY-DSD and DSD-free subjects. (A) Functional assays of the enzymatic kinetics of the WT (A49/L89; A49/V89) and mutated p.I161L (patient 5) variant. Each point represents the mean and standard deviation error bars of three independent replicates. (B) Structural analysis of the non-mutated SRD5A2 protein (WT) and the p.I161L variant (mutant). Structural modifications in amino acid side chain were visualized with PyMOL software. M_157_ =  Methionine 157; I_161_ =  Isoleucine 161; L_161_ =  Leucine 161; and Y_165_ =  Tyrosine 165.

To estimate the enzymatic activity of WT (rs523349 (L89V) variants and the mutated enzyme of steroid 5α-reductase type 2, different concentrations of T were used in sonicated cells (0.25–8.0 μmol/L). The WT enzyme (A49/L89) had a *K*_*m,app*_ of 0.72 ±  0.1 μM, *V*_*max,app*_ of 3819.3 ±  413.4 pmol/mg P/min, and catalytic eﬃciency *V*_*max*_*/K*_*m*_ of 5304 pmol DHT min^ − 1^ mg^ − 1^ (μmol/L)^ − 1^. In contrast, the WT A49/V89 polymorphism had a *K*_*m,app*_ of 0.7 ±  0.1 μM, *V*_*max,app*_ of 3367.6 ±  266.8 pmol/mg P/min, and *V*_*max*_/*K*_*m*_ of 4811 pmol DHT min^ − 1^ mg^ − 1^ (µmol/L) ^− 1^. The mutated p.I161L variant showed residual catalytic activity for DHT synthesis with *K*_*m,app*_ of 1.19 ±  0.1 μM, *V*_*m,app*_ of 688 ±  145.8 pmol/mg P/min, and *V*_*max*_*/K*_*m*_ of 578 pmol DHT min^ − 1^ mg^ − 1^ (μmol/L)^ − 1^ (Fig 4A).

Robetta and PyMOL software were used to obtain information on the 3D structural changes for WT and mutant SRD5A2 protein. Their structures and the locations of the substituted amino acid are indicated in Fig 4B. The p.I161L mutation appeared to retain the SRD5A2 protein’s 3D structure.

### *In-silico* analysis of SRD5A2 protein variants

The pathogenic functional effects of SRD5A2 protein variants were predicted and evaluated using PolyPhen-2 and PROVEAN software. The *in-silico* analysis indicated that sSNVs of C1–C3 (p.T81 = ; p.I198=) lacked a pathogenic association, so the variants were neutral substitutions. The nsSNVs in C4–C6 (p.A49T) predicted benign and deleterious effects. Protein variants in SRD5A2 from patients with steroid 5α-reductase type 2 deficiency were predicted as possibly damaging (Pos Dam) and probably damaging (Pb Dam) using PolyPhen-2. Bioinformatic analysis using PROVEAN showed neutral and deleterious effects (Table 1).

## Discussion

This report has described 9 non-synonymous gene variants of *SRD5A2* in patients with 46,XY-DSD resulting in steroid 5α-reductase type 2 deficiency. This genetic steroid disorder is increasingly being recognized for patients with hypospadias, microphallus, cryptorchidism, pseudovagina, inguinal testis, and an absence of axillary and pubic hair. This disorder is an autosomal, recessive, inherited disorder that is linked to male sex differentiation, and mothers or sisters of probands may carry the pathogenic variant without any clinical symptoms. Thus, this condition poses challenges in terms of genetic diagnosis and counselling.

Sanger sequencing has allowed an unprecedented level of genetic diagnostic success in such patients. This is especially notable when considering that for most patients with 46,XY-DSD, endocrine and clinical testing are insufficient. Therefore, an early genetic diagnosis can make future endocrine and imaging tests unnecessary, thereby reducing the financial, physical, and psychological burdens associated with this process [21].

Previous studies found that 8 of 9 nsSNVs of the *SRD5A2* gene were associated with deleterious effects on enzyme activity and steroid 5α-reductase type 2 deficiency [5,9,10]. In this regard, inactivating mutations increase the enzyme activity, display scarce or undetectable enzyme activity, or show roughly WT activity. Furthermore, in a compound-heterozygous patient (P5), genetic analysis identified a novel *SRD5A2*-nsSNV (c.481A > C; p.I161L), and its enzymatic activity resulted in a non-functional enzyme. Interestingly, in P6, co-existence of *SRD5A2* (p.F229S; this study and a previous study [5]) and *NR3C4* (p.G743E and a previous study [22]) gene mutations or nsSNVs may explain the incomplete regression of the Müllerian duct development and the complete androgen insensitivity syndrome (CAIS) phenotype with a 46,XY male karyotype.

Clinical evidence has shown that the p.G743E variant in the *NR3C4* gene can explain the completely female phenotype [22]. In patients with steroid 5α-reductase type 2 deficiency, the p.F229S variant in the *SRD5A2* gene has been reported to alter enzymatic activity, completely reducing the conversion of T to DHT [5]. Both findings suggest the possibility that T may be maintained as a substrate and aromatized to estradiol (E_2_; 1,3,5,[10]-Estratriene-3,17β-diol).

Previous studies have reported that estrogens can induce the formation of Müllerian ducts, and in particular, it has been observed that E_2_ has an inhibiting effect on *AMH* and *AMHR2* expression during follicular growth. Additionally, cryptorchidism impairs the functionality of Sertoli cells, which induces a low formation of AMH [23–27]. Cryptorchidism is associated with male infertility due to poor semen quality in adults. Furthermore, it impairs Sertoli cell function and possibly Leydig cell function as well [28,29]. The gene variants reported for *SRD5A2* (p.F229S) and *NR3C4* (p.G743E) could explain how the presence of Müllerian remnants in CAIS may be a steroid hormone-mediated defect.

Another interesting observation was the identification of 3 silent gene variants or sSNVs in the *SRD5A2* gene from DSD-free subjects or healthy subjects (C1–C3). In scientific reports, over 50 human diseases have been associated with synonymous mutations or sSNVs [30,31]. For instance, synonymous substitutions in the desert hedgehog (*DHH*) gene from DSD had a reproductive impact contributing to male infertility and gonadal dysgenesis [32]. There is also various evidence supporting the idea that the genetic diseases or traits related to synonymous mutations or sSNVs could be a consequence of another mechanism underlying non-silent synonymous mutations [33]. While the sSNVs found in DSD-free subjects were neutral, further experimental assays could be useful for investigating whether these silent gene variants have any effect on mRNA stability, splicing, or translation efficiency. Although sSNVs do not change the amino acid sequence of the protein directly, these substitutions might have important functional effects and could be of interest in genetic disorders, molecular evolution, and protein translation mechanisms. Genetic variants in the *SRD5A2* gene have been reported in various geographic locations worldwide [5]. In this context, it has been noted that missense SNVs or nsSNVs occur frequently in the *SRD5A2* gene and may be associated with steroid 5α-reductase type 2 deficiency. However, sSNVs have been reported less frequently. In this report, the sSNVs p.T81 = and p.I198 = from the Mexican population do not yield pathogenic consequences. Notably, these silent substitutions have been identified at a low frequency in other groups with Admixed American (rs757969706) and European/non-Finnish (rs28383064) ancestry, without any clinical significance (https://gnomad.broadinstitute.org/; accessed on December 2, 2024). As of the current date, these sSNVs have not been reported in literature or linked with clinical pathologies.

Multiple sSNVs have been reported in human gonad-determining genes (*WT1*, *NR0B1*, *LHX9*, and *ZNF275*) without pathogenic consequences [20]. Thus, several studies reflect the problem of the neutrality or functionality of the synonymous alleles and, specifically in this study, the sSNV linked to steroid 5α-reductase type 2 deficiency. This leads to questions regarding what genetic diseases could be susceptible to synonymous mutations or sSNVs. Another issue is related to what factors should characterize synonymous mutations or sSNVs (dependent on the structural domain or electrostatic DNA/protein interaction) that affect mRNA/protein structure or function by affecting pre-mRNA splicing, mRNA expression, stability, folding, micro-RNA binding, translation kinetics, and co-translational folding.

Answering these questions is difficult, although future studies could be developed with new strategies to identify the non-neutrality or neutrality of synonymous mutations and the genetic disorders in which these sSNVs may have biological and functional relevance. This study underscores the need for experimental assays for gene variants of synonymous significance to improve pathogenicity classification, which would allow us to better understand genotype-phenotype correlations. Hopefully, in the long term, this could also support the development of therapeutic strategies for patients with *SRD5A2*-associated 46,XY-DSD.

The last observation obtained in this study was the identification of an nsSNV (p.A49T) that is considered as a substitution without pathological consequences for *SRD5A2*-associated 46,XY-DSD. This heterozygous variant was identified in only three healthy subjects (C4–C6) and in one of the patients (P2). Molecular genetic evidence has demonstrated the existence of 2 coding polymorphisms (rs9282858: c.145G > A, p.A49T; rs523349: c.265C > G, p.L89V) in the *SRD5A2* gene [15,34]. These nsSNVs alter the protein coding region but do not impair phenotypical development. Nonetheless, the human *SRD5A2* p.A49T variant has been implicated in several clinical pathologies, such as hypospadias [35], prostate cancer [36], and breast cancer [37]. However, the implication of *SRD5A2*-linked genetic polymorphisms in cancer susceptibility and male sexual differentiation is complex and characterized by contradictory studies that have yet to be clarified. Similarly, the nsSNV (p.A49T) has been observed at a high frequency in groups of European/non-Finnish genetic ancestry (rs9282858), but its significance in relation to *SRD5A2*-DSD remains uncertain despite its benign classification (https://gnomad.broadinstitute.org/; accessed on December 2, 2024). These findings reveal that nsSNV (p.A49T) is not likely to be clinically significant in patients with 46,XY-DSD. However, the identification of this variation in both healthy controls and patients might suggest natural genetic variability with a very low allele frequency in the Mexican population.

Hormonal studies could contribute to understanding the genetic basis of SRD5A2-related disorders. Such studies can provide a more comprehensive picture of these findings. However, a limitation of this study was the exclusion of hormonal profiles primarily due to ethical limitations. Nevertheless, previous findings [38] in patients genetically diagnosed with steroid 5α-reductase type 2 deficiency have reported the typical pattern of normal T with decreased DHT and an elevated T/DHT ratio, which suggests a significant contribution of hormonal assays and genetics. Since this study excluded this hormonal profile, it was unable to address alternative diagnostic measures to account for this limitation. A limitation of the study was its small sample size (N =  6). However, previous reports have reported a low incidence of gene mutations within 46,XY-DSD of between 12% and 15% [39,40].

## Conclusion

Genomics and biochemistry offer an approach to showing the causality of *SRD5A2* SNVs. One non-synonymous single variant was crucial or sufficient to cause steroid 5α-reductase type 2 deficiency, and synonymous substitutions had a neutral effect on male sexual differentiation. Through integration of genetic polymorphisms in the *SRD5A2* gene with other omics data and functional description of causal mechanisms for genetic risk variants, steroid 5α-reductase type 2 deficiency genetics will continue to inform novel approaches to understanding the pathobiology of this genetic steroid disorder. It will also help in developing new strategies for clinical management, genetic counseling, and therapeutic approaches.

## Supporting information

S1 Checklist Human subjects research checklist. (DOCX)

S1 File Ethics approval letter-English. (DOC)

S2 File Ethics approval letter-Spanish. (PDF)

S1 Fig Fig 1 Original uncropped and unadjusted image. (DOCX)

S1 Table Supplementary table. (DOCX)

S2 Fig Fig 4 SRD5A2. (DOCX)
