# Supplementary material for: Novel non-synonymous and synonymous gene variants of SRD5A2 in patients with 46,XY-DSD and DSD-free subjects
Source: PLoS One. 2025 Mar 5;20(3):e0316497. doi: 10.1371/journal.pone.0316497 (PMC11882032; doi:10.1371/journal.pone.0316497)
Supplement: S1 File — (DOC) [file pone.0316497.s002.doc]

Mexico City, September 29, 2021

REG. CONBIOÉTICA-09-CEI-011-20160627

OFICIO No. MCONTROL-1507/2021

**DR. LUIS RAMOS TAVERA**

**PRINCIPAL RESEARCHER**

**DEPARTMENT OF REPRODUCTIVE BIOLOGY**

**INSTITUTO NACIONAL DE CIENCIAS MÉDICAS Y NUTRICIÓN SALVADOR ZUBIRÁN**

**AV. VASCO DE QUIROGA No. 15**

**COL. BELISARIO DOMÍNGUEZ SECCIÓN XVI**

**ALC. TLALPAN, C.P. 14080, CDMX**

In response to your letter dated August 30, in relation to the Clinical Research Protocol, entitled:

**“Insertion of mutations in the SRD5A2 gene and its impact on the catalytic properties of the enzyme” REF. 2613**

We inform you that we are aware of the current status of the study, and annual re-approval is authorized with validity until September 29, 2022.

Looking forward to hearing from you.

Sincerely,

**DR. CARLOS A. HINOJOSA BECERRIL DR. SERGIO C. HERNÁNDEZ JIMÉNEZ**

**PRESIDENT PRESIDENT**

**
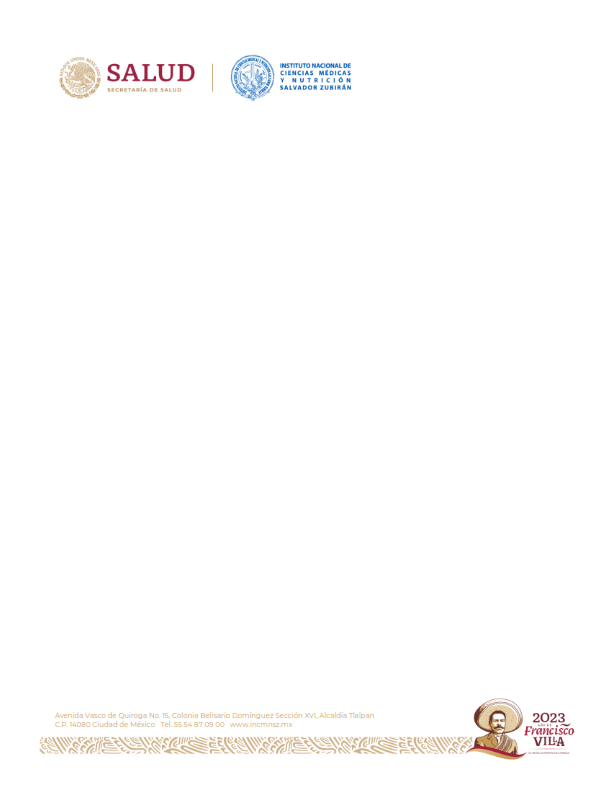
INVESTIGATION COMMITTEE RESEARCH ETHICS COMMITTEE**
