# Supplementary material for: Novel non-synonymous and synonymous gene variants of SRD5A2 in patients with 46,XY-DSD and DSD-free subjects
Source: PLoS One. 2025 Mar 5;20(3):e0316497. doi: 10.1371/journal.pone.0316497 (PMC11882032; doi:10.1371/journal.pone.0316497)
Supplement: S2 File — (PDF) [file pone.0316497.s003.pdf]

SALUD
SECRETARÍA DE SALUD

INSTITUTO NACIONAL DE CIENCIAS MÉDICAS Y NUTRICIÓN SALVADOR ZUBIRÁN

CIUDAD DE MÉXICO, A 29 DE SEPTIEMBRE DE 2021
REG. CONBIOÉTICA-09-CEI-011-20160627
OFICIO No. MCONTROL-1507/2021

DR. LUIS RAMOS TAVERA
INVESTIGADOR PRINCIPAL
DEPTO. DE BIOLOGÍA DE LA REPRODUCCIÓN
INSTITUTO NACIONAL DE CIENCIAS MÉDICAS Y NUTRICIÓN SALVADOR ZUBIRÁN
AV. VASCO DE QUIROGA No. 15
COL. BELISARIO DOMÍNGUEZ SECCIÓN XVI
DEL. TLALPAN, C.P. 14080, CDMX
P R E S E N T E

En respuesta a su oficio del pasado 30 de agosto, en relación al Protocolo de Investigación Clínica, titulado:

"Inserción de mutaciones en el gen SRD5A2 y su impacto sobre
Las propiedades catalíticas de la enzima"
REF. 2613

Le informamos que **se toma conocimiento** del estado actual del estudio, así mismo **su autoriza la re-aprobación anual con vigencia hasta el 29 de septiembre de 2022**.

Sin más por el momento, quedamos de Usted.

ATENTAMENTE,

DR. CARLOS ALBERTO AGUILAR SALINAS
PRESIDENTE
COMITÉ DE INVESTIGACIÓN

INS. NACIONAL DE CIENCIAS MEDICAS Y NUTRICION "SALVADOR ZUBIRAN"
29 SEP 2021
COMITÉ DE ÉTICA EN INVESTIGACIÓN

DR. SERGIO HERNÁNDEZ JIMÉNEZ
SECRETARIO
COMITÉ DE ÉTICA EN INVESTIGACIÓN

CAAS/SHJ/MARV

Escaneado con CamScanner
