# Supplementary material for: Novel non-synonymous and synonymous gene variants of SRD5A2 in patients with 46,XY-DSD and DSD-free subjects
Source: PLoS One. 2025 Mar 5;20(3):e0316497. doi: 10.1371/journal.pone.0316497 (PMC11882032; doi:10.1371/journal.pone.0316497)
Supplement: S1 Fig — (DOCX) [file pone.0316497.s004.docx]

46,XY-DSD patients with steroid 5α-reductase type 2 deficiency

N = 6

**A**

DSD-free subjects

N = 300

**gDNA isolation**

**Sample preparation**

**PCR amplification**

DSD-free subjects

N = 6 (C1–C6)

46,XY-DSD patients with steroid 5α-reductase type 2 deficiency

N = 6

**Sanger sequencing**

**Kinetic assays**

**3D structural substitutions**

**Pathogenic mutation prediction**

**Site-directed mutagenesis** **experiments**

**Experimental analysis**


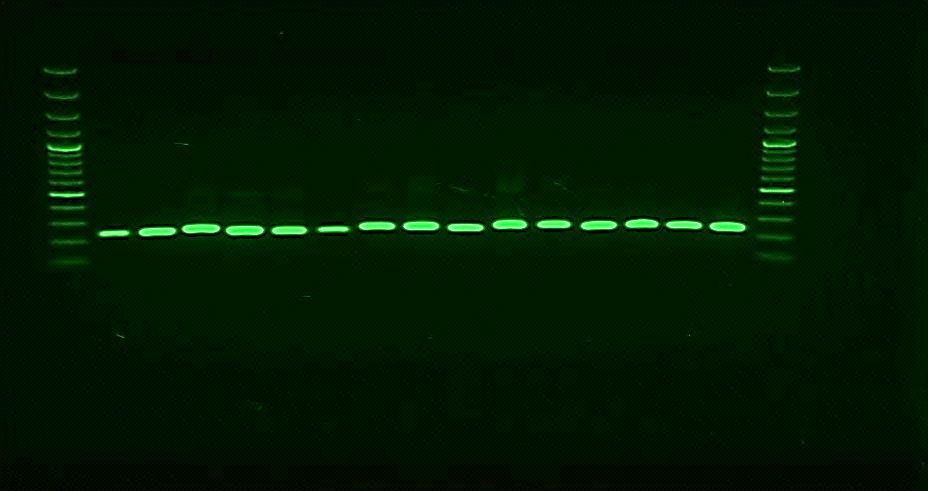


100 bp

**B**

**100 bp P1 P2 P2 P3 P3 P4 P5 P5 P6 C1 C2 C3 C4 C5 C6 100 bp**

214–248 bp

500 bp

**Fig. 1.**
