# Supplementary material for: Novel non-synonymous and synonymous gene variants of SRD5A2 in patients with 46,XY-DSD and DSD-free subjects
Source: PLoS One. 2025 Mar 5;20(3):e0316497. doi: 10.1371/journal.pone.0316497 (PMC11882032; doi:10.1371/journal.pone.0316497)
Supplement: S1 Table — (DOCX) [file pone.0316497.s005.docx]

**Supplementary table.** General characteristics of controls (N = 300).

| Controls | Ancestry | Controls (N) | Age (years) | Phenotype | Comments |
| --- | --- | --- | --- | --- | --- |
| Female | Mexican | 150 | 18–42 | Healthy subjects | Positive fertility |
| Male | Mexican | 150 | 18–42 | Healthy subjects | Positive fertility |
