# Supplementary material for: Novel non-synonymous and synonymous gene variants of SRD5A2 in patients with 46,XY-DSD and DSD-free subjects
Source: PLoS One. 2025 Mar 5;20(3):e0316497. doi: 10.1371/journal.pone.0316497 (PMC11882032; doi:10.1371/journal.pone.0316497)
Supplement: S2 Fig — (DOCX) [file pone.0316497.s006.docx]

| Table format:  **XY** | | **X** | **Group A** | | | **Group B** | | | **Group C** | | |
| --- | --- | --- | --- | --- | --- | --- | --- | --- | --- | --- | --- |
|  |  | Hours | p.L89 | | | p.V89 | | | p.I161L | | |
|  |  | **X** | **A:Y1** | **A:Y2** | **A:Y3** | **B:Y1** | **B:Y2** | **B:Y3** | **C:Y1** | **C:Y2** | **C:Y3** |
| **1** 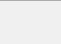 | Title | 0.00 | 0.000000 | 0.000000 | 0.000000 | 0.000000 | 0.000000 | 0.000000 | 0.000000 | 0.000000 | 0.000000 |
| **2** | Title | 0.25 | 717.456461 | 658.817231 | 547.295265 | 427.455344 | 332.611296 | 330.152159 | 201.523780 | 101.802940 | 84.356963 |
| **3** | Title | 0.50 | 1297.017550 | 2415.784510 | 1310.156550 | 749.735843 | 512.029193 | 584.091181 | 197.772847 | 154.093710 | 166.921686 |
| **4** | Title | 1.00 | 1627.251750 | 2240.806220 | 1923.027940 | 1547.543770 | 1213.061780 | 1021.962720 | 301.335973 | 202.016397 | 283.816638 |
| **5** | Title | 2.00 | 3203.295000 | 2957.171210 | 2355.729560 | 1368.272710 | 1997.041370 | 1852.270010 | 570.284341 | 455.808268 | 463.872544 |
| **6** | Title | 4.00 | 2686.304690 | 3137.912130 | 3688.804020 | 1568.564770 | 2403.588110 | 2278.155670 | 715.857428 | 501.914915 | 477.380489 |
| **7** | Title | 8.00 | 3321.272650 | 3510.306140 | 4502.008130 | 2826.325330 | 3294.252220 | 2492.502790 | 1246.043600 | 766.552657 | 1424.335540 |
